# Investigating the Evolution of Ingroup Favoritism Using a Minimal Group Interaction Paradigm: The Effects of Inter- and Intragroup Interdependence

**DOI:** 10.1371/journal.pone.0165974

**Published:** 2016-11-16

**Authors:** Kevin Durrheim, Michael Quayle, Colin G. Tredoux, Kim Titlestad, Larry Tooke

**Affiliations:** 1 Department of Psychology, University of KwaZulu-Natal, Pietermaritzburg, KwaZulu-Natal, South Africa; 2 Department of Psychology, University of Limerick, Limerick, Ireland; 3 Department of Psychology, University of Cape Town, Cape Town, Western Cape, South Africa; Peking University, CHINA

## Abstract

We investigated the effect of structural interdependencies between groups (especially inequality), and interdependencies between individuals on ingroup favoritism in minimal group situations. Previous research has attempted to determine whether ingroup favoritism is produced by categorization or intragroup interdependencies (reciprocation expectations), but recent literature suggests that it is not possible to tease these influences apart. We report two studies that investigate how ingroup favoritism evolves over time in social interaction. The levels of ingroup favoritism were affected by categorization and inequality, and the level of ingroup favoritism changed over time, increasing or decreasing depending on the nature of the initial intergroup structure. We conclude by providing two explanations for this change: emergent norms, and changes to the intergroup situation produced by interaction. Our experiments confirm the value of studying the evolution of minimal group behavior, especially for explaining why low status groups act to preserve intergroup inequalities.

## Introduction

The underlying causes of ingroup favoritism—or “parochial altruism”–have been of enduring interest to social psychologists, economists, and others seeking to explain the persistence of intergroup conflict, prejudice and inequality. However, researchers have seldom studied the evolution of ingroup favoritism.

In the first part of this article, we review experiments that have used the minimal group paradigm (MGP) to show that ingroup favoritism is a relational phenomenon, shaped by interdependencies between groups and individuals. Although relationships of interdependence evolve over time and in interaction, these temporal aspects of ingroup favoritism have only just begun to attract research attention. We then report the results of a program of experimental work that has sought to determine how the context and process of interaction can affect ingroup favoritism. We conclude by reflecting on our findings that interaction can promote both ingroup and outgroup favoritism, which can either accentuate or attenuate inequalities between groups.

### Intergroup Interdependence

The MGP eliminates social interaction, thus giving researchers sufficient control to study the underlying causes of ingroup favoritism. The benchmark studies were conducted by Tajfel and his colleagues in the early 1970s [1]. They randomly assigned twelve-year-old boys into groups and asked them to allocate monetary rewards to ingroup and outgroup members privately, without having met or interacted with any other participants. Although there was some evidence of fairness in the allocation, they found that participants also discriminated against outgroup members. This result has sometimes been interpreted as showing that ingroup favoritism is caused by social categorization [2].

However, this was not the significance that Tajfel and his colleagues drew from the studies [3, 4]. For them, the important finding was not that participants favored the ingroup, but that they were prepared to take less in absolute material terms in order to be relatively better off than the outgroup [2, 5]. Their findings argued against the primacy of economic explanations of social behavior. Here, experimental participants were seen to act against their naked economic interests, accepting less monetary reward for the ingroup in order to ensure that they were relatively better off than outgroup members. Turner et al. [6] suggested that there were social psychological interests at play, and Social Identity Theory was developed to explain the nature of these interests in terms of intergroup processes of categorization, comparison, and self-esteem:

“subjects define and evaluate themselves in terms of the imposed social categories, compare ingroup with outgroup in terms of the only available value dimensions (money, 'points,' evaluative scales) and seek positive distinctiveness for their own group by awarding it more money.”

(p.30)

We might characterize social identity theory as an intergroup interdependence theory of social behavior because it situates the phenomenon in the context of intergroup relations. Although Tajfel and his colleagues challenged Sherif’s economic explanation of prejudice, they endorsed his intergroup relations perspective. In his summer camp studies, Sherif [7, 8] had shown that prejudice could be created by setting groups in relations of economic competition, and could be eliminated by fostering intergroup cooperation for shared interest. In a similar vein, Tajfel and Turner [3, 4] argued that the positive distinctiveness motive would only result in ingroup favoritism under specified conditions of intergroup relations: It depended on whether the boundaries between groups were permeable, and whether the social status hierarchy was deemed legitimate and stable. Tajfel and Turner [4, 9] reviewed a number of cases in which low status group members manifested outgroup favoritism under conditions of social oppression, where individuals were unable to leave the group, and where the social status hierarchy was deemed stable and legitimate. The key to producing ingroup favoritism in the MGP was to create a particular kind of intergroup situation in which the boundaries were impermeable and status relations were unstable. These were the “certain conditions” under which Tajfel et al. [1] expected social categorization to lead to “intergroup behavior which discriminates against the outgroup and favors the ingroup.” (p.151)

Subsequent research has shown how other features of the intergroup situation can strengthen or weaken ingroup favoritism. Although low status groups demonstrate outgroup favoritism when the status differences are perceived to be stable and legitimate, they show ingroup favoritism when the status hierarchy is deemed illegitimate; and high status groups may respond to unfairness with compensatory favoritism toward low status groups [10, 11]. Similarly, ingroup favoritism is affected by ingroup and outgroup norms—e.g., competition versus fairness and loyalty versus equality—which define the nature of the intergroup relationship at any point in time [12, 13]. All these manipulations of the basic MGP support the intergroup interdependence perspective of social identity theory, namely, that categorization leads to ingroup favoritism only under certain conditions of intergroup relations.

### Intragroup Interdependence

Rabbie, Schot and Visser [14] argued that the minimal group studies conducted by Tajfel and his colleagues did not warrant the rejection of the economic explanation of ingroup favoritism. They argued that members of minimal groups were bound together by an “interdependence of fate” (p. 172) in addition to them belonging to distinct social categories. Consequently, interdependence and categorization were confounded, and it was possible that ingroup favoritism was driven by economic self-interest rather than social categorization. They argued that MGP participants attempted to secure most resources by cooperating with other ingroup members, believing ingroup members to be mutually dependent on each other and expecting ingroup members to reciprocate in turn.

The effects of reciprocation expectation on ingroup favoritism are now well-established. Locksley, Ortiz, and Hepburn [15] showed that ingroup favoritism in the MGP ceased when participants were given ‘feedback’ that in previous trials they were favored by outgroup rather than ingroup members. In contrast, ingroup favoritism could be strengthened if participants believed that they had been previously favored by the ingroup (see experiment 3 [16]; experiment 3 & 4 [15]; and experiment 1 [13]). Rabbie et al. [14] manipulated dependence and reciprocation expectations in a MGP experiment by telling participants that they would receive the money they were awarded by ingroup members only, outgroup members only, or from the allocations from both groups. Consistent with their expectations, they found that participants allocated most money to ingroup or outgroup members they perceived themselves to be most dependent on, even reversing ingroup favoritism in the outgroup dependence condition. Karp, Jin, Yamagishi and Shinotsuka [17] showed that ingroup favoritism in MGP disappeared when participants were paid a fixed sum by the experimenter. Gaertner and Insko [18] manipulated the dependence structure by giving participants unilateral control over the allocation of monetary rewards or by making their rewards dependent on the allocation by others; and the participants were either categorized into groups or not. Female participants favored the ingroup regardless of the dependence structure, whereas males favored the ingroup only in the presence of dependence.

Yamagishi and his colleagues have conducted a number of experiments to show that ingroup favoritism in the MGP depends on the expectation of ingroup reciprocation and is eliminated when reciprocation can be established directly with ingroup or outgroup members [19–21]. They had MGP participants play experimental two-player Prisoner’s Dilemma (PD) games in which individual payoffs depended on the decisions of both players. They would achieve the optimal collective outcome if both cooperated, but would do poorly if both defected. Individuals received the highest possible payoff if they defected but their partner cooperated, but would receive the lowest possible payoff if they cooperated but their partner defects. Ingroup favoritism was evident when participants cooperated with ingroup members but not outgroup members. Yamagishi and his colleagues argued that participants favored ingroup members in expectation of ingroup reciprocation, thus allowing cooperating ingroup members to achieve the highest collective payout. Hypothesized ingroup favoritism occurred in simultaneous games where players are thoroughly interdependent, each having to rely on the “generalized exchange belief” that ingroup members will reciprocate. However, this effect was extinguished in sequential games where participants made allocations as the first player, knowing that their allocation would be visible to the second player. Yamagishi and Mifune [21] argued that participants collaborated equally with ingroup and outgroup players in these sequential games in an effort to obtain resources through direct exchange rather than having to rely on “more uncertain generalized exchange”. (p. 21)

Simpson [22] studied ingroup favoritism in the last play of the sequential PD game. He argued that the first move (as studied by Yamagishi and colleagues) was affected by fear of exploitation, but the last move was entirely influenced by greed because the second player has a risk-free decision to cooperate for a lower payback or to exploit the cooperation of the first player for a greater payback. Whereas Yamagishi and colleagues showed that ingroup favoritism was switched off for first play fear responses, Simpson argued that ingroup favoritism would be apparent in second-play greed responses even though these were not affected by reciprocation expectations. His experiment supported this hypothesis, leading Simpson to conclude that ingroup favoritism is motivated by social identity processes associated with categorization and positive distinctiveness rather than interpersonal interdependencies.

In response to Simpson’s experiment, Yamagishi and Mifune [21] have refined their thinking about the nature of reciprocation expectancies. These have often been considered as vehicles for economic self-interest, but Yamagishi and Mifune [21] argue that they might primarily be motivated by a concern for personal reputation—which would nonetheless have self-interested economic spin-offs. They created a no-fear, greed-only situation in a Dictator Game in which the ‘dictator’ received the full allocation and then made a unilateral decision about how much to allocate to a recipient. They found that, in comparison to a condition where the recipient and receiver knew each other’s group identity (as studied by Simpson [22]), ingroup bias was extinguished when the dictator knew the group identity of the recipient but the recipient did not know the group status of the dictator. Dictators in this latter condition have a reduced risk of gaining a bad reputation from ingroup members, who expect to receive more from fellow ingroup members.

According to Yamagishi and Mifune [21], reciprocation expectations set up, and respond to, behavioral norms, which in turn affect individual reputations: “what is responsible for in-group favoring behavior is…the possible implications for one’s reputation, leading to exclusion from the group.” (p. 51) This is an alternative to economic interdependence theory. Ingroup favoritism arises through the dependence of the reputation of the actor to meet the normative expectations for behavior among ingroup (and outgroup) members rather than the dependence for economic profit on ingroup reciprocation and collusion.

Historically, interdependence theory had underpinned economic self-interest explanations of ingroup favoritism, but Yamagishi and Mifune have proposed a norm-based interdependence theory that is entirely compatible with social identity theory. In fact, this theory takes us full circle back to Tajfel’s early explanation of the behavior of MGP participants. Before he settled on the idea that ingroup favoritism was driven by self-esteem motives, Tajfel [23] argued that “human beings behave as they do because of the *social expectations* with which they enter an experiment—or any other social situation.” (p.72). He attributed ingroup favoritism to the human motive to act appropriately, and suggested that the MGP data be subject to “cultural analysis” of “the general norms and values that respondents were importing in order to deal with the novel situation.”(see [24], p.60). Cooperating with ingroup members was culturally deemed to be the right thing to do, as was intergroup fairness, which was so common that Tajfel [25] originally argued that allocations were “a compromise between fairness and favouring one's own group.” (p. 102). Being altruistic toward ingroup members is a way of creating a good reputation, leading to one being accepted into a network of generalized exchange, which is a precondition for acquiring positive identity and economic resources in social networks [21]. On the other hand, being fair to outgroups is a way of being an egalitarian citizen [26]. The value of these strategic goals in acting appropriately differs in importance, depending on the norms of the culture in which the experiment is undertaken, or the norms of the experimental situation, which can be manipulated, for example, by describing allocations as “points” or “payment” (ibid.).

### The Entangled Causes of Ingroup Favoritism

MGP research was initiated in the context of a debate about economic versus social psychological *causes* of ingroup favoritism. It has often taken the form of an either-or question: Is ingroup giving motivated by economic self-interest or by the desire to establish positive distinctiveness for members of a social category? The research cited above shows that group members tend to favor ingroup members in making resource allocations, but that this effect can be switched off or even reversed when interdependence with ingroup members is removed. There is thus widespread support for Rabbie et al.’s [14] claim that ingroup favoritism is motivated by economic self-interest, based on the expectation that ingroup members will reciprocate giving [27].

However, this research does not contradict Tajfel and Turner’s claim that social psychological motives underlie the phenomenon. There are a number of reasons to draw this conclusion. First, some research has found that ingroup favoritism is not influenced by the dependence manipulations, as was the case for Gaertner and Insko’s [18] female participants (see also [28]). Stroebe, Lodewijkx and Spears [29] found that ingroup favoritism in the MGP was influenced both by interpersonal interdependencies and by positive distinctiveness motives linked to social categorization. The importance of social identity dynamics is also underscored by research that has examined the way group membership in the MGP affects participants’ evaluation of others’ failure to show ingroup favoritism. The positive distinctiveness hypothesis is supported by the finding that MGP participants are less envious and more charitable toward ingroup members, even to the extent of forgiving their ‘misbehavior’ in terms of non-cooperation, outgroup giving, or lack of reciprocation [30, 31].

Collectively, this research raises questions about the wisdom of the “either-or” approach that has often been adopted in this field of investigation [29, 32]. The validity of interdependence versus social identity explanations of ingroup favoritism are often tested in designs that seek to manipulate outcome dependence and categorization orthogonally. A recent study by Platow, Grace and Smithson [33] suggests that this objective might ultimately not be attainable. They showed that categorization in the MGP was a sufficient condition to produce perceptions of interdependence. Together with the fact that interdependence induces categorization, this research indicates that social categorization and perceived interdependence are inextricably bound up together, with each being sufficient to produce the other. This causal entanglement is also evident in the reputational consequences of ingroup reciprocation expectations: Categorization affects behavioral norms, which are underlying resources for both material and reputational gain [21].

These causal entanglements suggests that research might profit from adopting a different strategy for studying ingroup favouritism. We recommend an evolutionary approach—one that seeks to show how the various underlying causes work together under different conditions of interdependence to stimulate or weaken ingroup favouritism.

### Interaction and the Evolution of Ingroup Favoritism

Our program of research does not propose a method for disentangling the causes of ingroup favoritism in the MGP. Rather, we study the way in which these strategic beliefs and motives function together in interaction over time and in different contexts to produce changing patterns of ingroup favoritism. Interdependence theory provides a useful starting point for our investigation because it situates ingroup favoritism in the context of social relationships: between groups in relations of status and power; and between individuals in relations of obligation and expectation. Sherif’s summer camp studies showed that ingroup favoritism could be produced by placing groups in relations of competition, and then eradicated when groups cooperated to attain a superordinate goal. However, these studies also showed that intergroup relationships were only partly under the control of the experimenters. The conflict and cooperation that emerged in interaction produced unanticipated outcomes, causing one study to be terminated early because of the violence that emerged and another study to be abandoned entirely when the groups of boys became allied against the experimenters [7, 34].

The MGP was developed to give experimenters more control by eliminating interaction between the groups and individuals in the experimental situation. The prohibition against interaction has meant, however, that thoroughly interactive phenomena such as interdependence and reciprocation have had to be carefully redefined and operationalized in non-interactive and static terms. In the research reviewed above, for example, outcome dependence has been manipulated by setting up various *expectations* for reciprocation. This has been done using allocation matrices where the experimenter either (a) informs participants that they received or did not receive tokens from ingroup or outgroup members in the past (e.g. [13, 15, 16]), or (b) defines allocation rules, manipulating whether rewards can come from either the ingroup, the outgroup, both the ingroup and the outgroup, or from the experimenter (e.g. [14, 17, 18, 29]).

Experimental games relax the restrictions on interaction that are enforced in MGP that use allocation matrices. Prisoner’s Dilemma games, for example, are interactional in the sense that outcomes depend on the actual decisions of both players [32]. However, these studies don’t facilitate true interaction either. In simultaneous games [20], the decisions of each player do not affect each other. First-player decisions in sequential games—the fear responses studied by Yamagishi and Kiyonari—are also not fully interactive, because they are not *responses* but one-off decisions. While second-player decisions in sequential games [22] are interactive responses to prior actions, the reason that they are stripped of all fear is because they constitute a final move—a last word—and will receive no response in return.

True interaction involves sequences of action, response, and reaction. Chen and Li [30] asked participants to allocate tokens to two other anonymous participants over a series of five rounds. They found that participants allocated almost equal amounts to two others who belonged to the same group—either ingroup or outgroup members—but they allocated more to ingroup members who were paired with outgroup members. The results also suggests that the difference between ingroup and outgroup allocation increased over the sequence of rounds. Although Chen and Li [30] did not test the difference between these slopes, the results suggest that ingroup favoritism strengthened over time. Just as repeated plays of the Prisoner’s Dilemma game can teach participants the benefits of cooperation [35], so too can ingroup favoritism evolve in interaction. Cacault, Goette, Lalive and Thoenig [36] asked members of three-person groups to contribute to public goods pools over a series of 5 rounds. When given the opportunity, group members acted aggressively by contributing to a commons that adversely affected outgroup earnings when they could (at no cost) have contributed to a commons that benefited both groups. Importantly, they also found a changing pattern of behavior over the sequence of rounds. Outgroup aggression in earlier rounds promoted altruistic giving in later rounds in comparison with the experimental condition that did not allow aggression. Dorrough, Glöckner, Hellmann and Ebert [37] asked members of two real world groups to play a sequence of 20 simultaneous PD games, first with 10 different members one group (ingroup or outgroup, counterbalanced), then with 10 different members of the other group. They found no evidence of ingroup favoritism at round 1, but found that ingroup favoritism increased over repeated contacts, and was linked to the expectation of ingroup reciprocation, especially in the shift between groups between rounds 10 and 11.

Chen and Li [30], Cacault et al. [36], and Dorrough et al., [37] studied ingroup favoritism over a sequence of rounds, and showed that subsequent behaviors may be influenced by prior actions. Nonetheless, these experiments lacked the responsive quality of true interaction. They did not give the recipients the opportunity to *respond* by making an allocation to the original allocator or the other recipients in the subsequent rounds. Such behavior would constitute a fully interactive network because at each point in a sequence of behaviors each actor has the opportunity to respond to all prior interactions, allowing the participants to collectively constitute the environment for subsequent interactions.

We have identified two studies that have investigated how participation in a fully interactive network affects ingroup favoritism. Goette, Huffman and Meier [38] studied military recruits in the Swiss army who had been randomly assigned into platoons and who spent weeks living together and interacting with members of their own platoon but without competition between platoons. In the wake of this interaction, participants played simultaneous Prisoner’s Dilemma games and were found to cooperate more willingly with ingroup members. Goette, Huffman and Meier [38] replicated the experiment but included a control condition of randomly assigned minimal groups that had no opportunity to interact. They found higher levels of cooperation among randomly assigned interacting than randomly assigned minimal groups. These results support other research that shows that interaction promotes a sense of groupness, which can affect behavioral outcomes [39, 40].

The within-group interaction among platoon members in these experiments extended over time and allowed all group members to interact with each other, responding to previous actions. The network was fully interactive, but with uncontrolled and undocumented interaction, and so we have no way of knowing what features of interaction produced the outcomes of interest, or how prior interactions affected subsequent ones. The interaction was a ‘black box’ of unknown content—but which as a whole was shown to affect social identity, collective behavior, and ingroup favoritism.

In recent years, computer simulations using simple artificial agents have gone some way to open the black box of interaction. They have shown how group-based phenomena such as ethnocentrism can emerge over time, as agents make simple decisions to either defect or cooperate with ingroup and outgroup agents [41]. Such simulations situate social behavior in fully interactive networks, where all agents may interact with all others, and where action at any point in time is a response to what went before and constitutes the interactional context for subsequent behavior. Simulations have helped to identify conditions that promote the evolution of ingroup favoritism—including lower cost of cooperation, a greater number of groups, greater communication between agents, and an optimal—midrange—rate of migration between groups [42–46].

These software agents might make simple, rule-governed and self-interested decisions, stripped of the complexity of human social life, but the simulations lay bare the evolutionary dynamics of ingroup favoritism. These fully interactive social contexts show that the “dynamic nature of bias results from complex social network interactions which play a central role in human societies.” ([43] p.1). These networks of interactions simultaneously reflect and produce interpersonal and intergroup interdependencies, which evolve in interaction to constitute the “nature of the relations between groups” which Sherif [7] and Tajfel [47] both recognized was the foundation of ingroup favoritism.

### Hypotheses and Expectations: Intragroup and Interpersonal Social Interdependence

**Fig 1:**
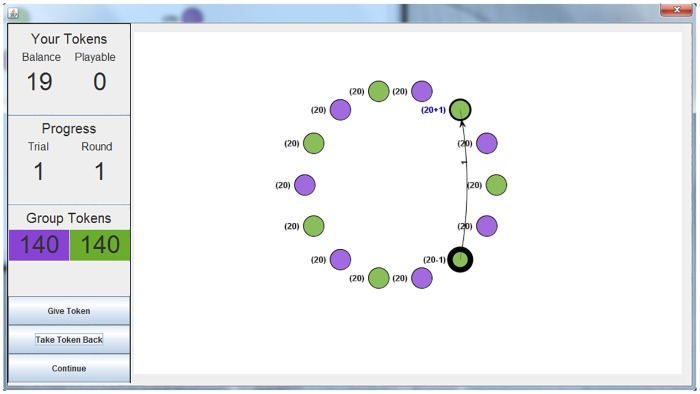
Illustration of a VIAPPL environment, where avatars are circles, the current participant is bold, and each player’s token balance is indicated alongside the avatar.

We used the VIAPPL experimental platform to facilitate and document interaction and its effects in the MGP (see www.viappl.org). We created exchange games in VIAPPL in which each participant was represented by an avatar located in a sparse virtual arena in which all other participant avatars were visible (see Fig 1). Each participant owned a balance of tokens, and the games were played by a single rule: each participant was required to give one token to any other player in the arena during each round of play. Token exchanges were made by clicking on any avatar in the arena, and ‘giving’ the token. Each act of giving added one token to the recipient’s fortune (or balance), and subtracted one token from the giver’s balance. At the conclusion of each round all the exchanges and final token balances were represented on the screen for all participants to see.

The games reported in this article all had 14 players and were played over 40 rounds. We manipulated the initial starting balances to create equal and unequal situations. In addition, we manipulated group condition by creating two types of games: one in which all players were undifferentiated individuals, and another in which participants were assigned to one of two minimal groups, each identifiable by a distinct avatar color. We manipulated inequality orthogonally to group membership, creating equal and unequal individual and group games. Finally, in Study 1 only, we replicated each condition with a competition, fairness or no-norm (control) prime. The design thus manipulated intergroup interdependencies, allowing us to investigate the exchange practices of equal status, high status, and low status groups and individuals in different normative climates. We report all measures, manipulations, and exclusions in these studies, and determined sample size in advance of data collection by ensuring that we had sufficient number of game replications in each condition of the design.

Our dependent variable was defined by the target of giving. We recorded whether each player in each round gave their token to an ingroup member, an outgroup member, or to themselves. Given the 40-round game structure, each form of giving potentially ranged from 0 to 40, and there were dependencies between the three forms of giving which together summed to 40. Ingroup favoritism was indexed by higher ingroup and lower outgroup giving. Even though the avatars in the individual condition were not represented as distinguishable group members, to compare allocation practices in the individual and group conditions we treated each individual as having the group identity of the individual in the same position in the arena in the group condition.

In the light of the substantial literature on ingroup favoritism in the MGP we propose the following general hypotheses for the effects of the experimental manipulations:

H_G_1. There will be more ingroup giving and less outgroup giving in the group than individual condition.
H_G_2. In the inequality condition there will be more “compensatory” ingroup giving and less outgroup giving among low status groups, and there will be more “compensatory” outgroup giving and less ingroup giving among high status groups. We expect compensatory responses because our inequality condition is illegitimate in the sense that it is imposed by experimenters and not ‘earned’ by the participants [11]. In contrast, because status relations in the equality condition are insecure, we anticipated competitive ingroup favoritism in the equal status games.
H_G_3. Competition primes will strengthen ingroup favoritism whereas fairness norms will weaken ingroup favoritism.

We anticipated that these effects would interact with each other by strengthening ingroup favoritism when they are aligned or by weakening ingroup giving when they are not aligned. For example, compensatory ingroup giving of low status groups and outgroup giving of high status groups will be further strengthened by the fairness prime, whereas the competitive ingroup favoritisms in the equality condition will be strengthened by the competition prime.

It is only in Round 1 that the experimental situation is fully defined by the experimenters, as is usually the case in MGP experiments. As the games unfold, the exchanges that occur in each round begin to change the environment of individual and group balances and differences. Moreover, a sense of sociality emerges as ties are made between individuals and groups and players respond by reciprocating (or not), acting selfishly, or by ingroup or outgroup favoritism. Thus, each replication of a game with the same set of starting conditions will not be expected to produce the same outcome. This is the evolutionary process that is our primary interest.

In this exploratory study we investigate how ingroup favoritism evolves over time and in response to different starting experimental conditions. We propose the following temporal hypotheses about the evolution of ingroup favoritism in social interaction.

H_T_1. Static model without interdependence: Behavior is prescribed by a fixed underlying cause or set of causes (e.g., economic self-interest or positive distinctiveness motive) that assumes a constant strength from round one. The motives and beliefs underlying such interaction are similar to the fixed “happiness rules” that inform agent behavior in simple simulations. Although no one explicitly defends this model, it is implicit in most research that only makes use of one-off observations to identify *the* cause—in an either-or fashion—of ingroup favoritism. This is the model, without interdependence, that John Turner spent a lifetime arguing against [5].
H_T_2. Interpersonal interdependence model of emerging norms (Monotonic change model). This is similar to the static model, but with normative strengthening of tendencies. We expect monotonic change as initial behavioral tendencies strengthen over time as they acquire normative status.
H_T_3. Intergroup interdependence model: This is similar to the interpersonal interdependence model, but we expect different emergent norms under different conditions of intergroup relations as predicted by Social Identity Theory [3, 4].
H_T_4. Dynamic interdependence model (non-monotonic change model). This model anticipates that behavioral imperatives and norms are (1) shaped by nature of the relations between groups and individuals within groups, and (2) change over time as they are shaped by prior actions. Intergroup hierarchies and the legitimacy and stability of intergroup relations are not stable ([11] p.8), neither are reciprocation expectancies; so we also anticipated effects where initially dominant responses might strengthen, weaken or even reverse over time.

H_T_1 is opposed to the other temporal hypotheses, and while we don’t expect to find evidence for H_T_1, we did not hypothesize which of H_T_2, H_T_3 and H_T_4 we expected to identify, or which would be most prominent.

## Study 1

### Method

#### Sample

The sample consisted of 1005 student volunteers (490 female, 476 Male; 855 Black, 27 White, 49 Indian, 32 Coloured) from the University of KwaZulu-Natal, South Africa. The mean age of the participants was 20.59 years (SD = 2.16 years). All participants provided written informed consent to participate in the study, which had been approved by the Human Sciences Research Ethics Committee of the University of KwaZulu-Natal.

#### Design

Participants played 40-round games in which they were required to allocate one token per round to any of the participants in the game, including themselves. We paid all participants a standard incentive of R20 for their participation irrespective of their final token balance, meaning that the tokens had a purely symbolic value. The requirement to allocate one and only one token per round resulted in a structural dependency between ingroup-, outgroup-, and self-giving, yielding compositional data in which each participant’s outgroup-, ingroup-, and self-giving summed to one in each round, and 40 over the rounds.

Four experimental factors were operationalized as follows:

#### Group condition

Participants were randomly assigned to play either as undifferentiated individuals or as members of visually distinctive groups. Assignment to minimal groups was random but was done via a painting preference task in which participants indicated their preference for a painting by Klee or Kandinsky. In the individual condition, player avatars were notionally assigned to the same groups as in the group conditions, but participants were not aware of this group membership because no visual or textual indicators of group membership were given. In the individual inequality condition, this underlying group membership was made evident by the difference in starting balance between the high status and low status individuals.

#### Equality condition

Equality condition was manipulated orthogonally to group condition. Half the games in the group and individual conditions were equal status games and the other half were unequal status games. In the equal status games, all participants were assigned the same starting token balance, namely 30 tokens. In unequal status games, participants assigned to the low status condition were allocated 20 tokens, whereas participants assigned to the high status condition were allocated 40 tokens. This manipulation created a second experimental factor, Equality Condition, with three levels—equal status (n = 503), low status (n = 251) and high status (n = 252).

#### Norm condition

Immediately before starting the game, the participants completed an adaptation of the verbal memory task developed by Hertel and Fiedler [12, 48], which primed either no norm, fairness or competition. Neutral words, such as computer, car and tree, were used throughout the three primes. Only neutral words were used in the control (no norm) condition. Competition was evoked through words such as opponent, hostility, struggle; whereas the fairness prime included words such as balance, justice, and democratic.

#### Time

We partitioned the data for each game into 5 waves of 8 rounds each, and summed the tokens given to self, ingroup, and outgroup in each wave.

When crossed, these factors resulted in a 12-cell design, where each of the 12 cells represented a VIAPPL game to be played by 14 participants under a specific set of conditions, with the game in each cell replicated six times. This resulted in a total of 72 VIAPPL games in the study with 1005 participants (three participants failed to complete the game), making a total of 5025 observations of ingroup- outgroup- and self-giving (1005 participants by five 8-round waves).

### Results

**Fig 2:**
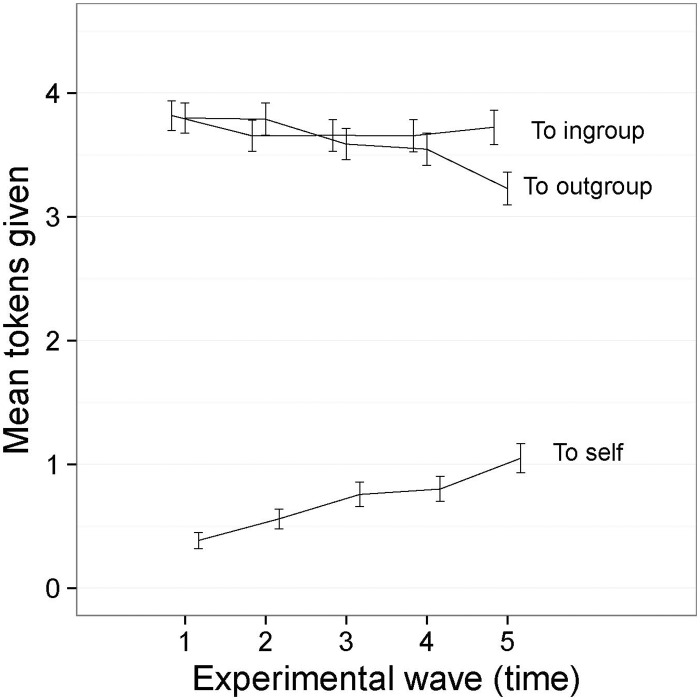
Mean frequency of ingroup-, outgroup- and self-giving over time (Study 1, symbolic tokens). I bars are independent 95% confidence intervals.

Counts of ingroup-, outgroup-, and self-giving could each range from 0 to 8 (and jointly summed to 8) in each wave. Ingroup- and outgroup-giving were approximately normally distributed, but self-giving was positively skewed and zero-inflated. The frequency of self-giving was relatively low compared to ingroup- and outgroup-giving, but increased steadily over time (See Fig 2). There was no difference in the frequency of ingroup- and outgroup-giving at the start of the games, but outgroup-giving declined over time whereas the rate of ingroup-giving held constant. It appears as though self-giving partially replaced outgroup-giving but not ingroup-giving in later rounds of the games.

We planned to use multi-level modelling (NLME and LME4 packages in R) to account for the nesting of the three kinds of token giving (1) in waves, (2) repeated by individuals, who, in turn, were (3) nested in games. However, it proved infeasible to analyse our compositional, longitudinal data with multi-level models. We thus decided to analyze ingroup versus outgroup giving independently from self-giving, eliminating the compositional aspect of the data, and allowing us to use multilevel models. We operationalized ingroup favoritism as the proportion of non-self-giving that was ingroup giving:

$$Ingroup\;Favoritism=\frac{ingroup\;giving}{ingroup\;giving+outgroup\;giving}$$

This removes the longitudinal trend of rising self-giving—which was evident in all cells of the design –from the dependent variable, and in the process rectifies the dependency in the compositional nature of the data. It also eliminates the problem of skewness and zero-inflation that was apparent with the self-giving data. Because self-giving was zero-inflated, we reduced self-giving to a binary variable for each individual in each wave to index whether any self-giving apparent in that wave. We then conducted a binomial regression (using generalized linear mixed modelling) to predict self-giving from the main effects, Time, Group Condition, Equality Condition, and Norm Condition. Only Time significantly predicted self-giving (z = 10.46, p< .0001). Models that included interaction terms would not converge, perhaps due to the zero inflation. This analysis together with an inspection of descriptive data showed that self-giving increased over time for all the experimental conditions, and that the mean level of self-giving was the same in all experimental conditions.

Preliminary analyses of ingroup favoritism indicated that the random intercept models significantly improved model fit, in comparison with the null model (AIC_null_ = 1183.19). We first included only games as a random factor, which improved fit over the null model (AIC = 740.68; LRatio = 444.51, df = 1, p <. 0001), and then we tested the model that included individuals and games as random factors, which also improved fit significantly over the null model, and produced a much smaller value of AIC (AIC = -130.79, LRatio = 1317.97, df = 2, p <. 0001). We then added the four fixed effects to the model, and compared the model with random intercepts (AIC = -273.89) against the model with random intercepts and random slopes (AIC = -394.71), treating time as a random effect. Adding random slopes improved the fit (LRatio = 128.83, df = 4, p <.0001). We decided on the basis of this preliminary testing to include the three nested random effects subjects, time, and games, in further multilevel models, estimating random effects for intercepts and slope terms.

Because of the complexity of the model and the size of the dataset, we used a simple model with a single Time by Group condition interaction to experiment with different correlation structures to take the autocorrelation between the repeated observations into account. The AR1 correction improved the model fit (LRatio = 25.96, p <.0001) and improved the distribution of the residuals, which were approximately normally distributed with the double nesting in place.

We then tested the full model by adding the 4-way interaction and all lower order interactions into the mixed effects multilevel model described above. The interactions of a higher order than two-way were not significant, nor were any of the effects involving norm condition. We thus ran the final model that included the two significant two-way interactions—Time by Group Condition and Time by Equality Condition—and the main effects (see Table 1). The model achieved a fit no worse than the fit provided by the model with all the two way interactions present (AIC = -374.70 vs -296.76, LogLik = 203.35 vs 176.38, LRatio = 14.65, p > 0.26), and also no worse than that provided by the model with all the three-way interactions (AIC = -374.70 vs -222.29, LogLik = 203.35 vs 151.15, LRatio = 32.68, p > 0.11). Estimates of AIC, and Log Likelihood were obtained for each model using restricted maximum likelihood algorithms (REML), but model comparisons (the likelihood ratio) were computed using full maximum likelihood (ML). For our model with the two two-way interactions, we also computed pseudo-R^2^ values for the overall model, following the approach of Nakagawa and Schielzeth [49], which returned marginal and conditional R^2^ values of 0.10 and 0.50 respectively (the marginal R^2^ represents the proportion of variance explained by the fixed effects, and the conditional R^2^ the proportion explained by the random effects; it is clear that in our model both fixed and random effects are substantial, but random effects account for a lot more variation than the fixed effects). Table 1 reports both general level Anova results for the model (Type II SS), and model fixed effect coefficients. The coefficients have associated 95% confidence intervals reported, as well as approximate standardizations of the coefficient values (they are scaled by the residual standard deviation). These results are intended to provide a sense of the substantive size of the effects, rather than just their statistical significance.

**Table 1:** ANOVA and coefficient summary of multilevel model of the 2013 data, symbolic token exchange.

Fixed Effects	Model coefficients							
Source	χ^2^	Df	p <	Source	B	Lower CI	Upper CI	b / rsd
Experiment Round (Time)	3.94	1	0.048	Intercept	0.52	0.49	0.55	
Group	50.33	1	0.001	Time	0.02	0.01	0.03	0.08
Equality	124.81	2	0.001	Group	-0.10	-0.06	-0.13	-0.46
Group x Time	3.63	1	0.057	Equality: high vs equal	-0.10	-0.06	-0.14	-0.47
Equality x Time	18.95	2	0.001	Equality: low vs equal	0.15	0.10	0.19	0.70
*Random effects*				Time X Group	-0.01	-0.02	0.00	-0.05
Source	Int. (SD)	Slope (SD)	R	Time X Equality_high	0.00	-0.01	0.02	0.02
Time | Game	0.03*	0.01*	0.33	Time X Equality_low	-0.02	-0.04	-0.01	-0.11
Time | Game | Particip	0.1*3	0.03*	-0.28					
Residual	0.21							

Note: CI = 95% confidence interval. rsd = residual standard deviation. Int. = intercept.

**Fig 3:**
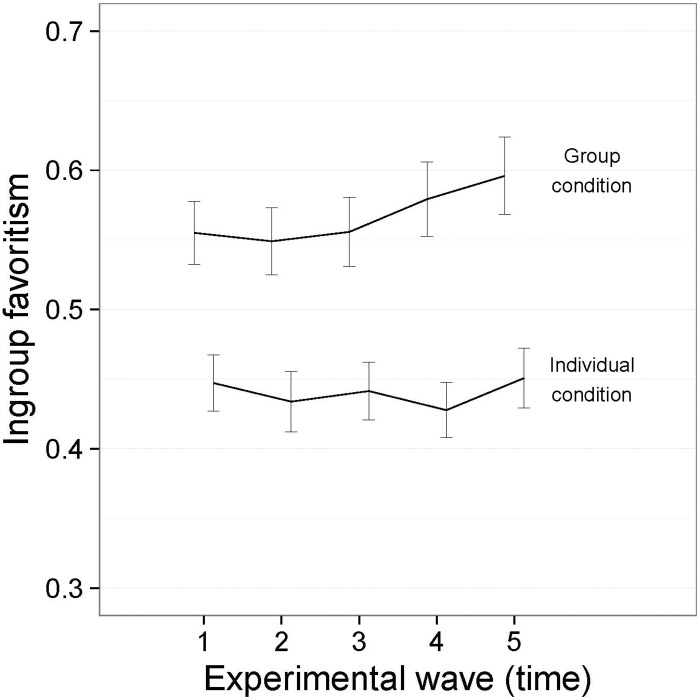
Interaction between Group Condition and Time (Study 1, symbolic tokens). I bars are independent 95% confidence intervals.

The main effect for Time indicates that, overall, there was a slight tendency for ingroup favoritism to increase over time. The interaction between Time and Group Condition shows that this effect was apparent among players who were assigned to minimal groups, but not among players in the individual condition—see Fig 3, where we report means and 95% confidence intervals around the means.

Participants in the minimal groups started out making more ingroup versus outgroup allocations than participants in the individual condition, and this difference strengthened over the course of the games. Given that there is no significant difference in the mean level of self-giving in the individual and group conditions, these results suggest that self-giving replaces outgroup giving in the minimal groups, but replaces both ingroup and outgroup giving in the individual condition.

**Fig 4:**
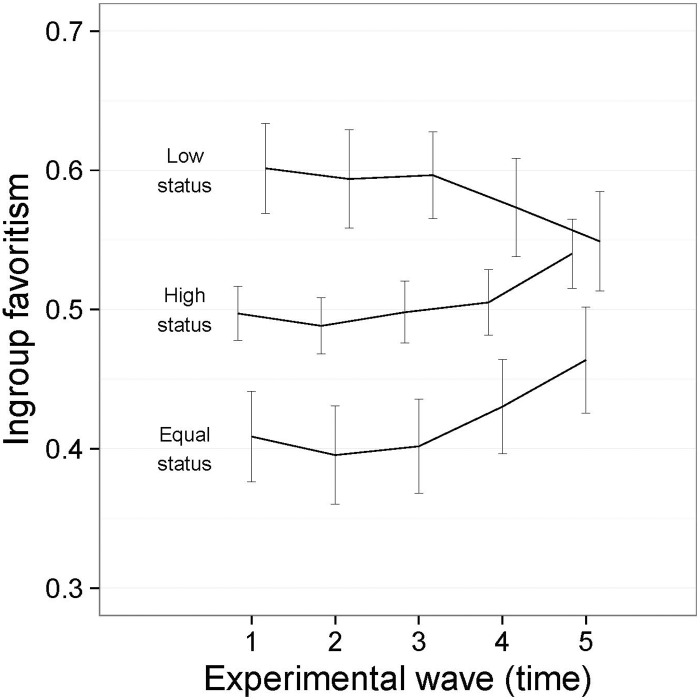
Interaction between Equality Condition and Time (Study 1, symbolic tokens). I bars are independent 95% confidence intervals.

The main effect for Equality Condition (see Fig 4) reflected the higher levels of ingroup favoritism among the low status group than the equal status group and the lower levels of ingroup favoritism among the high status group than the equal status group. High status participants—who began with 40 tokens—allocated more tokens to the low status participants than to their own group, whereas those who started the game with 20 tokens allocated more to the ingroup than the outgroup. This is the pattern of compensatory allocation we expected in the context of an illegitimate inequality; and the absence of a three-way interaction indicated that the pattern was apparent equally in the group and individual conditions.

The interaction between Equality Condition and Time provides very strong support for the need for an evolutionary approach to the study of ingroup favoritism. The pattern of compensatory giving to the low status groups is very strong among the high and low status groups at the beginning of the game, but declines for both groups in the second half of the game. Perhaps this decline can be anticipated for high status groups for whom ingroup favoritism begins to play a stronger role as the initial inequality begins to decline. It is surprising, however, that low status groups increase the rate of giving to the high status groups in later rounds even when the intergroup inequality persists. It appears as though the generosity from the high status outgroup in earlier rounds promotes reciprocation that serves to preserve the inequality. This is the case despite the fact that inequality persists throughout, with the mean round balances after each wave being as follows: 151:266, 161:256, 170:248, 177:241, and 180:237.

### Discussion

The results of Study 1 supported hypotheses H_G_1 and H_G_2, but not H_G_3. Ingroup favoritism was stronger in the group than the individual condition (H_G_1), and we observed the pattern of compensatory outgroup giving among the high status groups and compensatory ingroup giving among the low status groups in the inequality condition (H_G_2). There was no effect for the norm condition manipulation (H_G_3), either because the prime was not powerful enough to induce norms, or because competition and fairness norms have no bearing on behavior in this context. In sum, these findings provide strong support for the social identity theory of ingroup favoritism among minimal groups. Ingroup favoritism was apparent in the allocation of tokens that had no monetary value and was much more frequent than self-giving. This pattern of giving appeared to be motivated by the desire to be in the ‘winning group’ irrespective of material rewards. Previous MGP research has been criticized for excluding self-interest [28], but we found evidence of ingroup favoritism in the presence of self-giving; and in fact, the increasing incidence of self-giving in later rounds, appeared to strengthen ingroup favoritism. Overall, Study 1 supported the intergroup interdependence model of social identity theory, which anticipates that ingroup favoritism effects will be contingent upon the situation. Accordingly, we were able to create a situation of ingroup favoritism in the equal status condition, strengthen this tendency for low status groups, and reverse it for high status groups.

The finding that that the two significant interactions involved Time supports our argument for the importance of an evolutionary approach to studying ingroup favoritism. The data show that ingroup favoring norms tend to increase over time. This supports the hypothesis H_T_2 that proposed that initial behavioral tendencies would strengthen as they acquired normative status. This trend was apparent both in the case of ingroup favoritism and self-giving. The results for the inequality condition support the intergroup interdependence hypothesis, H_T_3, which proposed that different emergent norms would evolve under different conditions of intergroup relations.

## Study 2

Study 2 was a replication and extension of Study 1, but we used monetary rather than symbolic rewards, and excluded Norm Condition, which was found to have no impact on the allocations in Study 1. In this replication of the experiment, conducted in 2014, each token represented one South African Rand (approximately US $ 0.09), and the participants were told that they would receive as payment the token balance they achieved at the end of the game. Given these material outcomes, we anticipated greater self-giving and ingroup favoritism as group members used ingroup affiliation and reciprocation expectations to increase their material rewards.

### Method

#### Sample

The sample consisted of 224 student volunteers (97% female, 125 Male; 209 Black, 3 White, 3 Coloured, 6 Indian) who each participated in one of 16 14-player experimental games. The mean age of the participants was 20.47 years (SD = 2.28 years). All participants provided written informed consent to participate in the study, which had been approved by the Human Sciences Research Ethics Committee of the University of KwaZulu-Natal.

#### Design

During each of the 40 rounds of each game, participants were required to give one token to any of the participants in the game, including themselves. Three experimental factors were operationalized as follows:

#### Group condition

Participants were randomly assigned to play either as undifferentiated individuals (8 games) or as members of visually distinct groups (8 games). Assignment to minimal groups was random but was done via a dot estimation task by which participants were informed that they would be assigned to a group of ‘over estimators’ or ‘under estimators’. In the individual condition, player avatars were assigned to the same groups as in the group conditions, but participants were not aware of this group membership because no visual or textual indicators of group membership were given (Except where it could be inferred from different starting token balances in the inequality condition).

#### Equality condition

Equality condition was manipulated orthogonally to group condition. Half the games in the group and individual conditions were equal status games and the other half were unequal status games. In the equal status games, all participants were assigned the same starting token balance, namely 20 tokens. In unequal status games, participants in the low status group were assigned 10 tokens, whereas participants in the high status group were assigned 30 tokens. This manipulation created a second experimental factor, Equality Condition—with three levels, equal status (n = 112), low status (n = 56) and high status (n = 56).

#### Time

We partitioned the data for each game into 5 waves of 8 rounds each, and summed the number of tokens given to self, ingroup, and outgroup in each wave.

### Results

The total number of allocation decisions made in the experiment was 8960 (16 games x 14 participants x 40 rounds), which resulted in 1120 8-round waves.

The distribution of ingroup-, outgroup-, and self-giving across all the 8-round waves revealed a stronger ingroup favoritism preference than in Study 1. Self-giving remained negatively skewed and very strongly zero inflated. Ingroup-giving was negatively skewed and outgroup-giving was positively skewed, reflecting the preference for ingroup-giving over outgroup-giving.

**Fig 5:**
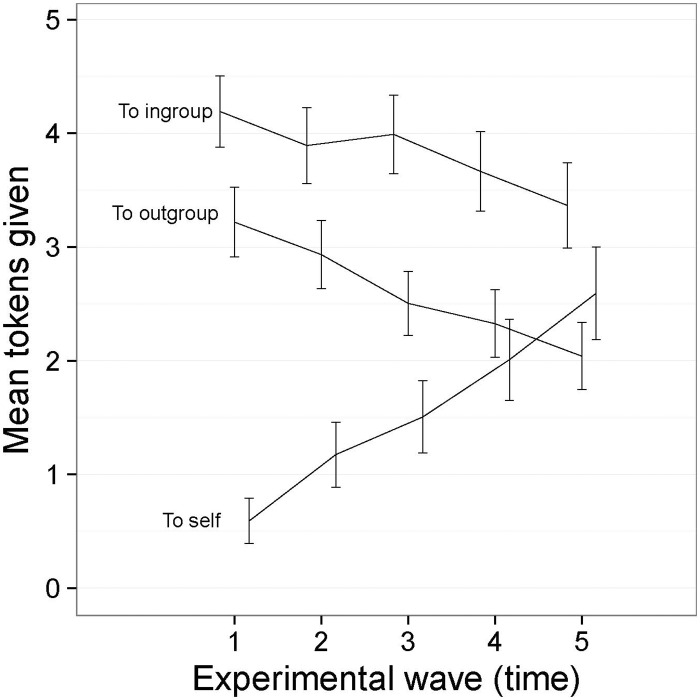
Mean frequency of ingroup-, outgroup- and self-giving over time (Study 2, monetary tokens). I bars are independent 95% confidence intervals.

Fig 5 reports the changing frequency of ingroup-, outgroup-, and self-giving over time. The differential rate of ingroup- versus outgroup-giving expands in the second wave and then persists throughout the games. In contrast, the rate of self-giving increases monotonically and sharply over time, and it would appear as though self-giving replaces both ingroup- and outgroup-giving. In Study 1, by contrast, self-giving replaced outgroup- but not ingroup-giving.

We used multi-level modelling (NLME and LME4 packages in R) with maximum likelihood estimation to analyze the data while taking onto consideration the nesting of token giving (1) in waves, (2) repeated by individuals, who, in turn, were (3) nested in games. We computed ingroup favoritism in the same manner as we did in Study 1, as the ratio of ingroup-giving to ingroup- plus outgroup-giving, thus removing self-giving from ingroup favoritism. The skewness in the data that we report above was fairly small, and when we assessed the ML model residuals, these were approximately normal, suggesting that the skewness did not adversely affect model results.

Preliminary analyses indicated that random intercept models significantly reduced the AIC in comparison with the null model (null AIC = 767.25). We first included only games as a random factor, which provided a better fit than the null model (AIC = 583.64; LRatio = 185.61, df = 1, p < .0001) and then we tested the model that included individuals and games as random factors, which provided a better fit too than the null model, with a lower AIC value (AIC = 351.51, LRatio = 419.74, df = 2, p <.0001). We then added the three fixed effects to the model, and compared the model with random intercepts (AIC = 309.41) to the model with random intercepts and random slopes (AIC = 271.78). Adding random slopes improved the fit (LRatio = 45.62, df = 4, p <.0001). The AR1 correction for autocorrelation improved the model fit (LRatio = 9.51, df = 1, p <.002) and the residuals were approximately normally distributed with the double nesting in place.

We then tested the full model with the three-way interaction and all lower order interactions and main effects. The three-way interaction was not significant (χ^2^ =.34, df = 2, p > .84); neither was the two-way interaction between Group Condition and Equality Condition (χ^2^ = 2.91, df = 2, p > .23). Our final model thus included the two-way interactions between Time and Group Condition and Time and Equality Condition and the three main effects (AIC = −294.74, LogLik = -131.37) (see Table 2). Estimates of AIC, and Log Likelihood were obtained for each model using restricted maximum likelihood algorithms (REML), but model comparisons (the likelihood ratio) were computed using full maximum likelihood (ML). For our model with the two two-way interactions, we also computed pseudo-R^2^ values for the overall model, following the approach of Nakagawa and Schielzeth [49], which returned marginal and conditional R^2^ values of 0.22 and 0.59 respectively (it is clear that in our model both fixed and random effects are substantial, but random effects account for a lot more variation than the fixed effects). Table 2 reports both general level Anova results for the model (Type II SS), and model fixed effect coefficients (from the REML model). The coefficients have associated 95% confidence intervals reported, as well as approximate standardizations of the coefficient values (they are scaled by the residual standard deviation). These results are intended to provide a sense of the substantive size of the effects, rather than just their statistical significance.

**Table 2:** ANOVA and coefficient summary of multilevel model of the 2014 data, monetary token exchange.

Fixed Effects	Model coefficients							
Source	χ^2^	Df	p <	Source	B	Lower CI	Upper CI	b / rsd
Experiment Round (Time)	0.86	1	0.35	Intercept	0.66	0.59	0.73	
Group	46.18	1	0.001	Time	0.01	-0.01	0.04	0.05
Equality	38.74	2	0.001	Group	-0.16	-0.25	-0.08	-0.67
Group x Time	5.66	1	0.02	Equality: high vs equal	0.19	0.09	-0.14	0.78
Equality x Time	17.25	2	0.001	Equality: low vs equal	-0.22	-0.32	-0.12	-0.91
*Random effects*				Time X Group	-0.03	-0.06	-0.01	-0.14
Source	Int. (SD)	Slope (SD)	R	Time X Equality_high	-0.04	-0.07	-0.01	-0/16
Time | Game	0.00*	0.02*	0.22	Time X Equality_low	0.03	-0.00	0.06	0.13
Time | Game | Particip	0.11*	0.03*	0.41					
Residual	0.25							

Note: CI = 95% confidence interval. rsd = residual standard deviation. Int. = intercept.

Overall test of random effects χ^2^(4) = 34.3, p < 0.001. Phi of correlation structure = 0.23.

* p < 0.05 for random effects

**Fig 6:**
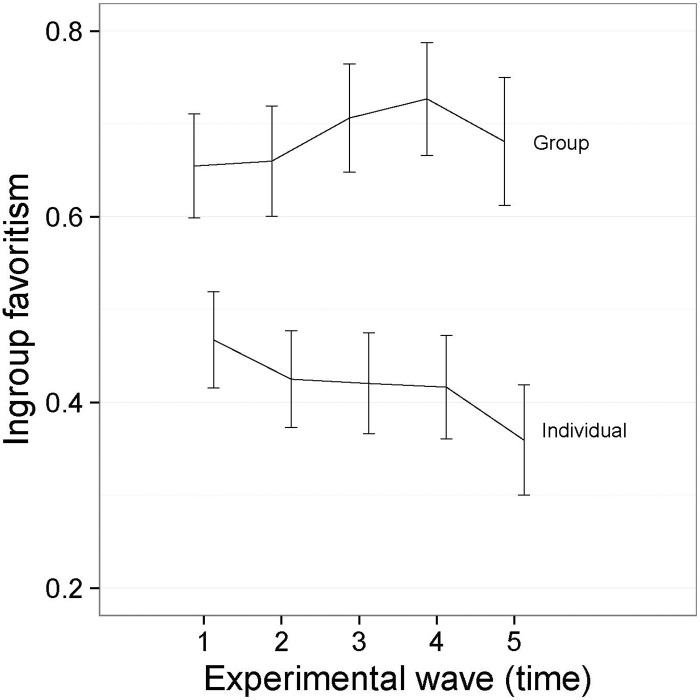
Interaction between Group Condition and Time (Study 2, monetary tokens). I bars are independent 95% confidence intervals.

**Fig 7:**
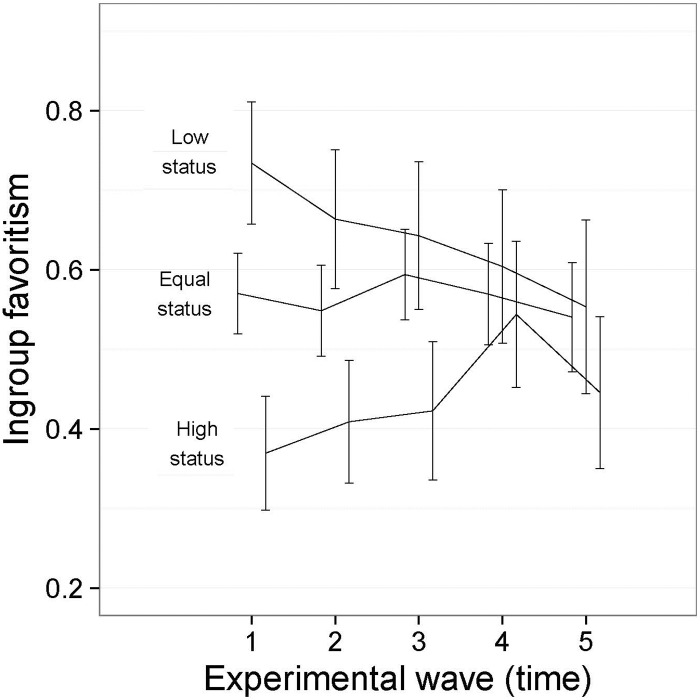
Interaction between Equality Condition and Time (Study 2, monetary tokens). I bars are independent 95% confidence intervals.

An inspection of the figures that represent how Group Condition (Fig 6) and Equality Condition (Fig 7) interact with Time shows why the main effect for Time is not significant: Ingroup favoritism changes in different directions in the different experimental conditions. Overall participants showed more ingroup favoritism in the group than in the individual condition (t = 4.08, df = 14, p < 0.002), but ingroup favoritism also tended to increase over time in the group condition but decrease over time in the individual condition. Self-giving tends to replace ingroup-giving in later rounds of the individual condition but tends to replace outgroup-giving in the group condition. Both graphs show a dip in ingroup favoritism in the final period as self-giving begins to replace ingroup-giving. This decline is most likely what game theorists refer to as an end-game effect, which is common in finitely repeated games [50]. In this case, it appears that behavior became more overtly selfish as the game approached its end because self-giving guaranteed additional tokens and the prospect of being sanctioned for selfishness—by not receiving tokens from ingroup members—decreased.

The main effect for Equality Condition replicate the finding that the low status group showed higher ingroup favoritism than the equal status group (t = 4.50, df = 206, p < .0001), whereas the high status group showed lower levels of ingroup favoritism than the equal status group (t = 3.88, df = 206, p < .0001). This confirms our expectation that the high status group would engage in compensatory outgroup favoritism given that our manipulation of inequality was illegitimate. This difference between the high and low status groups decreased over time because the high status group showed more ingroup favoritism over time, whereas the low status group showed less ingroup favoritism over time. In the final period, however, the high status group showed sharply less ingroup favoritism as self-giving replaced ingroup- but not outgroup-giving. These results provide strong evidence of an intergroup interdependence effect. The high status groups started the games with a higher rate of outgroup- versus ingroup-giving, but this generosity decreased over time as the initial illegitimate advantage reduced but before the inequality was eliminated. The low status group members initially favored their own group-players, but this ingroup favoritism also decreased over time as which helped to preserve the intergroup inequality in later rounds (the mean round balances for the high and low status groups after each wave are as follows: 191:89; 177:103; 166:115; 161:119; 157:123). By the forth wave there is not difference ingroup favoritism between the high and lost status groups, because both groups are acting in a way that preserves the inequality.

### Discussion

Study 2 found higher levels of ingroup favoritism and self-giving than Study 1, consistent with the idea that these strategies were vehicles for leveraging economic gain. Overall, however, the pattern of significant main and interaction effects was remarkably similar to that found in Study 1. The results provided support for the hypothesis that ingroup favoritism would be stronger under the group than individual condition (H_G_1). The results for inequality conformed the compensatory giving we expected in the context of an illegitimate inequality manipulation (H_G_2). The results confirm the importance of the effect of group-based inequalities on social interdependencies.

The same interactions with Time were apparent in Study 2 as were evident in Study 1. The difference between the levels of ingroup favoritism between the individual and group condition increased over time; and the levels of ingroup favoritism by the high and low status groups converged over time. These findings support the interpersonal and intergroup interdependency hypotheses (H_T_2 and H_T_3) as was the case in Study 1.

## Overall Discussion

Minimal group research has been deeply interested in the role that social interdependencies play in promoting ingroup favoritism. Tajfel and his colleagues [1] initially focused on the way intergroup interdependencies lead group members to act against their immediate economic self-interest. In the unstable and equal status intergroup relations of their original minimal groups studies they observed that participants declined greater ingroup rewards in absolute terms to ensure that they were relatively better off than the other group. Group interests—which would later be theorized by social identity theory—appeared to have replaced the economic interests that Sherif had argued were necessary for prejudice. However, Tajfel and Turner [3, 4] anticipated that there would be situations where ingroup bias would be absent or even reversed. In particular, they argued that members of low status groups would show outgroup favoritism when their low status was deemed legitimate or when the social system was deemed stable.

It soon became clear that interpersonal and intragroup interdependencies also affected reward allocations in the MGP. Participants tended to favor those who had favored them in the past or were likely to favor them in the future. These findings provided the basis for an alternative explanation of ingroup favoritism in the MGP. Because participants expected higher levels of reciprocation from ingroup members than outgroup members, they show ‘ingroup favoritism’ in allocating to ingroup members in the hope that they will ultimately gain when ingroup members allocated to them in return (cf. [51]).

A great deal of MGP research has been devoted to teasing apart social identity processes from interdependency processes in minimal group situations. Is ingroup favoritism produced by categorization and identification or is it produced by reciprocation expectations? Recent evidence and a number of reviews have reached the conclusion that both processes affect behavior in the MGP and, moreover, that the two processes are themselves mutually interdependent [27, 32]. Categorization is a sufficient condition for perceptions of interdependence, and interdependence is a sufficient condition for categorization. Consequently, further attempts to tease these apart experimentally might not be fruitful.

One of the consequences of this avenue of investigation has been to fix attention on ingroup favoritism rather than, say, on the conditions that promote outgroup favoritism. This has positioned MGP research within the social psychological tradition of work that addressed the ‘prejudice problematic’[52]. In contrast to economists who tended to see the positive aspects of “ingroup altruism” social psychologists focused on the problem of prejudice that was exemplified by ingroup favoritism in the MGP.

The present research makes two novel contributions to this tradition of social psychological scholarship. Our first contribution was explicit right from the beginning of this research project: we have investigated how ingroup favoritism evolves over time. To achieve this goal we put in place two essential conditions for the evolution of social life, namely, interaction between social actors, and a temporal context in which behaviors are allowed to influence each other and begin to create emergent contexts for subsequent interaction. We developed VIAPPL as an experimental platform for an evolutionary social psychology (see viappl.org). Our minimal group studies in this interactive context confirm the expectations that categorization promotes ingroup favoritism and that an illegitimate status hierarchy can promote compensatory outgroup favoritism among high status groups and ingroup favoritism among low status groups.

In addition, the present research shows how the strength of ingroup favoritism may change over time. One reason for this is because acts of ingroup-, outgroup- and self-giving may acquire normative status and acceptability as they are picked up by other participants during the course of the interaction. This appeared to be the case for self-giving, which was a dispreferred, low frequency behavior at the start of the games, but which became more popular over time. In addition to emerging norms, there was a second way in which interaction produces change: behavior that responds to initial structural conditions can change those conditions thereby weakening the rationale for that behavior. This was evident in the weakening pattern of compensatory ingroup and outgroup giving of the low and high status groups in both studies. This pattern of allocation gradually reduced the intergroup inequality which, in turn, weakened the need for compensation and reduced the outgroup favoritism among high status group members and ingroup favoritism among low status group members. These results indicate that compensatory ingroup favoritism of low status groups and outgroup favoritism of high status groups is quickly extinguished. This pattern of compensatory intergroup behavior that has been evident in non-evolutionary minimal group studies and in early rounds of the present studies does not appear to persist over time. Both high and low status groups changed their exchange behavior in later rounds to preserve the inequality that was initially deemed illegitimate.

In addition to promoting an evolutionary approach, this study has made a second contribution to the MGP literature. Although our data confirms much of what we already know about ingroup favoritism, it also sheds light on some of the processes that may undermine social change orientation of minority group members. Social identity theory ascribes outgroup favoritism among minority group members to a stable and oppressive social system which prevents the cultivation of cognitive alternatives, and which fosters self-depreciating responses among minority group members [9, 53]. Jost and Banaji [54] argued that these were system justifying responses that helped oppressed groups to “adapt to the unjust or unpleasant realities that appear to be inevitable.” ([55] p.146)

In contrast to both of these accounts, the present research proposes interactional reasons for the outgroup favoritism of minority group members. In both studies, the initial ingroup favoritism of the low status groups decreased over the course of the game to the point where it was equal to the ingroup favoritism of equal and high status groups despite the fact that substantial inequalities remained between the groups. Members of the poor group began to increasingly favor the wealthy group in making allocations. Why would they act against their own group interest in this way? One possibility is that they did so to reciprocate for the favor the high status group had shown them in earlier rounds of compensatory outgroup favoritism of the high status group. Another possibility is that they hoped to elicit future reciprocation and benefit by giving to wealthy outgroup members. Perhaps the lowered inequality and the early compensatory exchanges led the participants to view remaining equality as more legitimate than the initial unearned inequality that the experimenters had imposed.

Whatever the reason, the results suggests that intergroup interdependencies created by inequality can affect the kind of interpersonal interdependences that social actors attempt to set up. Generous treatment from high status group members may build expectations of fair treatment, which may in turn promote outgroup favoritism among low status groups in the face of ongoing inequality, even when the expectations of fair treatment from this high status group are not met (cf. [56]).

In the article we have advanced a minimal group interaction paradigm (MGIP) in order to study the evolution of ingroup and outgroup favoritism in interactive exchange networks. VIAPPL reintroduces social interaction into the context of social psychological experiments, marking a return to the interactive experiments of an earlier generation (Sherif, Asch, Milgram, and Zimbardo). However, in contrast to these earlier interactive experiments, the form of interaction in VIAPPL is strictly controlled and observed so that it is possible to document the evolution of social life and its outcomes. In this article, however, our progress in studying inter- and intra-group interdependencies has been limited by our use of aggregate data and statistical techniques that assume independence of observations, and that thus partial out the real interdependencies in the data at a local level (i.e. between actors). In future work we plan to study local structural interdependencies using the methods of network analysis.

## Supporting Information

S1 File Data dictionary 2013 and 2014 data files. (XLSX)

S2 File Vpldata2013wider-201697.Rdata. (CSV)

S3 File Vpldata2014revwider-2016104.Rdata. (CSV)
